# A randomized controlled trial of eicosapentaenoic acid and/or aspirin for colorectal adenoma prevention during colonoscopic surveillance in the NHS Bowel Cancer Screening Programme (The seAFOod Polyp Prevention Trial): study protocol for a randomized controlled trial

**DOI:** 10.1186/1745-6215-14-237

**Published:** 2013-07-29

**Authors:** Mark A Hull, Anna C Sandell, Alan A Montgomery, Richard FA Logan, Gayle M Clifford, Colin J Rees, Paul M Loadman, Diane Whitham

**Affiliations:** 1 Leeds Institute of Biomedical & Clinical Sciences, St James’s University Hospital, Leeds LS9 7TF, UK; 2 Nottingham Clinical Trials Unit, C Floor South Block, Queens Medical Centre, Nottingham NG7 2UH, UK; 3 Nottingham Digestive Diseases Centre, Queen’s Medical Centre, University of Nottingham, Nottingham NG7 2UH, UK; 4 South Tyneside NHS Foundation Trust, South Shields, Tyne and Wear NE34 0PL, UK; 5 Institute of Cancer Therapeutics, University of Bradford, Bradford BD7 1DP, UK

**Keywords:** Aspirin, Colorectal adenoma, Colorectal cancer, Eicosapentaenoic acid, Omega-3 polyunsaturated fatty acid

## Abstract

**Background:**

The naturally-occurring omega (ω)-3 polyunsaturated fatty acid (PUFA) eicosapentaenoic acid (EPA) reduces colorectal adenoma (polyp) number and size in patients with familial adenomatous polyposis. The safety profile and potential cardiovascular benefits associated with ω-3 PUFAs make EPA a strong candidate for colorectal cancer (CRC) chemoprevention, alone or in combination with aspirin, which itself has recognized anti-CRC activity. Colorectal adenoma number and size are recognized as biomarkers of future CRC risk and are established as surrogate end-points in CRC chemoprevention trials.

**Design:**

The seAFOod Polyp Prevention Trial is a randomized, double-blind, placebo-controlled, 2 × 2 factorial ‘efficacy’ study, which will determine whether EPA prevents colorectal adenomas, either alone or in combination with aspirin. Participants are 55–73 year-old patients, who have been identified as ‘high risk’ (detection of ≥5 small adenomas or ≥3 adenomas with at least one being ≥10 mm in diameter) at screening colonoscopy in the English Bowel Cancer Screening Programme (BCSP). Exclusion criteria include the need for more than one repeat endoscopy within the three-month BCSP screening period, malignant change in an adenoma, regular use of aspirin or non-aspirin non-steroidal anti-inflammatory drugs, regular use of fish oil supplements and concomitant warfarin or anti-platelet agent therapy. Patients are randomized to either EPA-free fatty acid 1 g twice daily or identical placebo AND aspirin 300 mg once daily or identical placebo, for approximately 12 months. The primary end-point is the number of participants with one or more adenomas detected at routine one-year BCSP surveillance colonoscopy. Secondary end-points include the number of adenomas (total and ‘advanced’) per patient, the location (left *versus* right colon) of colorectal adenomas and the number of participants re-classified as ‘intermediate risk’ for future surveillance. Exploratory end-points include levels of bioactive lipid mediators such as ω-3 PUFAs, resolvin E1 and PGE-M in plasma, urine, erythrocytes and rectal mucosa in order to gain insights into the mechanism(s) of action of EPA and aspirin, alone and in combination, as well as to discover predictive biomarkers of chemopreventive efficacy. The recruitment target is 904 patients.

**Trial Registration:**

Current Controlled Trials ISRCTN05926847

## Background

### Colorectal cancer chemoprevention

The scientific and clinical rationale for prevention of colorectal cancer (CRC) is firmly established [1]. CRC prevention strategies include population screening, endoscopic surveillance of high-risk groups, chemoprevention (the use of drugs, vitamins or other food supplements), and health education leading to lifestyle modifications such as weight loss and physical activity [1].

The long natural history of human ‘sporadic’ colorectal carcinogenesis, during which tumor initiation and benign adenoma (or polyp) growth precede transformation into a clinically apparent malignant adenocarcinoma (or cancer) over a number of years, has been the basis for CRC prevention strategies aimed at detection and removal of asymptomatic colorectal adenomas in healthy individuals (either directly by colonoscopy- or flexible sigmoidoscopy (FS)-based screening [2], or indirectly via colonoscopy prompted by faecal occult blood testing [1]). Colonoscopic polypectomy has been demonstrated to reduce CRC mortality [3]. However, risk reduction was approximately 50% in the US National Polyp Study analysis [3] and ‘interval’ CRC during screening and surveillance programmes is increasingly recognized [4]. Therefore, there is still an unmet clinical need for safe and effective CRC chemoprevention, in combination with existing screening and surveillance programmes [5].

### Candidate CRC chemoprevention agents

There are several potential CRC chemoprevention agents including non-steroidal anti-inflammatory drugs (NSAIDs), hormone replacement therapy and micro-nutrient administration, e.g., folic acid, vitamin D [6]. The largest body of evidence supports the use of the NSAID aspirin for CRC chemoprevention [7]. However, aspirin has not yet been advocated for primary or secondary CRC chemoprevention due to continuing uncertainty about the optimal daily dose (different trials have reported efficacy of either high- (>300 mg) or low-dose (<100 mg) aspirin [7]) and the absence of a clearly defined at-risk population, in whom benefit would outweigh the small risk of gastro-intestinal and intra-cerebral bleeding associated with aspirin [7].

Combination therapy is widely recognized as a promising strategy for CRC chemoprevention, particularly if the combination of agents has other beneficial effects [8]. Omega (ω)-3 polyunsaturated fatty acids (PUFAs) are attractive candidate ‘natural’ CRC chemoprevention agents for evaluation alone and in combination with aspirin, given that both agents also have proven cardiovascular benefits and are already widely prescribed together following myocardial infarction [9].

### Omega-3 polyunsaturated fatty acids

Polyunsaturated fatty acids are important components of the normal diet. Two classes of PUFAs, ω-6 and ω-3 PUFAs, are classified as essential in that they cannot be readily synthesized in the human body and so must be obtained from dietary sources [10]. The principal ω-3 PUFAs are C20:5 eicosapentaenoic acid (EPA) and C22:6 docosahexaenoic acid (DHA), which are found predominantly in oily, cold-water fish such as mackerel, having entered the food chain following synthesis by plankton [10]. However, in ‘western’ diets, ω-6 PUFAs predominate including C20:4 arachidonic acid, which is the main substrate for cyclooxygenase (COX) enzymes [10].

### Anti-CRC activity of ω-3 PUFAs

There is a strong body of pre-clinical evidence that ω-3 PUFAs have anti-CRC activity [11]. Recently, high dietary intake of marine-derived ω-3 PUFAs has been associated with reduced colorectal adenoma risk [12]. Equivocal benefit of ω-3 PUFA intake in some human observational studies may be related to methodological difficulties measuring ω-3 PUFA or fish intake [10] and the fact that dietary ω-3 PUFA exposure may not be sufficient for consistent anti-CRC activity in individuals consuming moderate amounts of fish (a portion of oily fish 2–3 times per week only provides the equivalent of approximately 500 mg per day of EPA and DHA combined) [10].

A 500 mg gastro-resistant capsule formulation of EPA as the free fatty acid (FFA) is now available for administration of large amounts of EPA, up to 2 g daily. EPA is released from the capsules and absorbed maximally in the small intestine, thereby minimizing gastrointestinal side-effects. EPA as the free fatty acid (EPA-FFA) is significantly better absorbed than EPA in the usual ethyl ester or triglyceride forms [13].

EPA-FFA has recently been demonstrated to reduce intestinal adenoma multiplicity by 79% in the *Apc*^*Min/+*^ mouse model of familial adenomatous polyposis (FAP) [14]. This study led to a Phase III double-blind RCT of the effect of treatment with EPA-FFA 2 g daily for 6 months on rectal polyps in patients with FAP [15]. This trial has provided the first definitive evidence of chemopreventive efficacy of EPA in humans with a net decrease in adenoma numbers and a cumulative reduction in adenoma size of 22.4% and 29.8%, respectively, between the EPA and placebo arms [15]. The percentage reduction in polyp burden was similar to the anti-neoplastic activity previously observed in FAP patients treated with the selective COX-2 inhibitor celecoxib [16], a drug which was subsequently demonstrated to prevent ‘sporadic’ colorectal adenomas in a polyp prevention trial [17].

### Mechanisms of the anti-neoplastic activity of EPA and aspirin

The precise mechanism(s) by which EPA and aspirin have anti-CRC activity are not fully understood [10,18]. However, it is currently accepted that, even though these agents are likely to act via both COX-dependent and -independent mechanisms, modulation of COX activity plays an important role in their anti-neoplastic effects. EPA and aspirin are both potent inhibitors of COX-1 but they alter COX-2 activity in different ways leading to production of different bioactive lipid mediators, including PGE_3_ (EPA) and 15*R*-HETE (aspirin) [18]. Aspirin irreversibly acetylates the COX enzymes leading to conversion of EPA to 18*R*-hydroxyeicosapentaenoic acid (18*R*-HEPE) and then trihydroxy-EPA, also known as resolvin (Rv) E1, which has potent anti-inflammatory activity [19]. Therefore, there is a biochemical basis for a potential interaction between EPA and aspirin. The available clinical evidence suggests that the cardiovascular effects of EPA and aspirin are simply additive based on the accumulated evidence of extensive use of dual therapy in cardiology patients [9]. However, there is evidence for both an additive and synergistic relationship between aspirin and ω-3 PUFAs from independent *ex vivo* human platelet aggregation studies [20,21].

### A polyp prevention trial using patients requiring ‘high risk’ colonoscopic surveillance

The adenomatous polyp, particularly the ‘advanced’ lesion (≥10 mm diameter, with tubulo-villous/villous histology or with high-grade dysplasia), is an established surrogate biomarker of CRC risk and has been used consistently as a primary colonoscopic end-point in multiple short-term (up to 3 years) CRC chemoprevention trials [17,22].

Previous polyp prevention trials have recruited patients that are roughly equivalent to ‘intermediate risk’ patients in the Bowel Cancer Screening Programme (BCSP), which uses the same definition as the British Society of Gastroenterology adenoma surveillance guidelines, i.e., 3–4 adenomas detected or at least one adenoma >10 mm in diameter [23]. The three-year total adenoma recurrence rate in these polyp prevention trials varied between 25–50% in the placebo arm [1,7,17,22]. Recruitment of ‘high risk’ (defined as ≥5 small adenomas or ≥3 adenomas, with at least one being ≥10 mm in diameter) BCSP patients undergoing surveillance colonoscopy one year after the last complete screening colonoscopy [23] capitalizes on a higher adenoma recurrence rate (>60%; unpublished data) at an earlier (12–15 month) time-point, thus providing sample size benefits and reduced trial duration. Previous concerns about the use of an approximate one-year end-point in polyp prevention trials have been allayed by the observation that adenoma outcomes at one year have consistently mirrored those reported at later time-points [22].

Another methodological consideration relates to the possible effect of ‘missed’ adenomas rather than ‘new’ lesions detected during short-term colonoscopic assessment of ‘recurrence’. Preliminary data from the South of Tyne and Tees BCSP Centres have demonstrated that one or more adenomas (that can be assumed to be ‘missed’) were detected in 36% of 44 high risk individuals who underwent a check colonoscopy to assess a polypectomy site within three months of the index colonoscopy, compared with a 66.2% adenoma recurrence rate at one year. This large difference in adenoma detection between 3 and 12 months supports the supposition that there is *de novo* adenoma growth over a 12 month period and is mirrored by data from a similar American study [24]. Moreover, 86% of the ‘recurrent’ adenomas at one year in the South of Tyne and Tees cohort were small, non-advanced adenomas (rather than ‘advanced’ lesions), which would be expected if the majority of adenomas detected at this time-point represented *de novo* adenoma growth. In practice, short-term colonoscopic ‘recurrence’, even in expert hands, represents a combination of ‘new’ and ‘missed’ adenomas. Therefore, chemopreventive efficacy observed in RCTs is likely to be a combination of polyp prevention and regression, a concept that has been readily accepted in ‘proof-of-principle’ FAP RCTs [15,16]. Importantly, the reduction in adenoma ‘recurrence’ in the aspirin polyp prevention RCTs [22] has predicted the longer-term effect of aspirin on CRC incidence [7], confirming the utility of adenoma recurrence as a surrogate biomarker of CRC risk.

From 2013, FS screening (termed Bowel Scope in the BCSP) will be offered to all people aged 55 years, with self-referrals accepted up to the age of 60. National roll-out across England is expected by 2016. Any patient undergoing FS in the Bowel Scope programme, who has a polyp ≥10 mm, ≥3 adenomas, an adenoma with a tubulovillous or villous component, an adenoma with high-grade dysplasia, or in whom polypectomy is not appropriate at screening FS, will be referred for full screening colonoscopy. Adenoma outcomes will be summated from the FS and subsequent full colonoscopy for the purposes of risk stratification for future surveillance colonoscopy. Therefore, FS screening provides another pathway for identifying ‘high risk’ individuals who require one-year surveillance colonoscopy.

### Aims of the seAFOod Polyp Prevention Trial and hypotheses to be tested

The primary aim of the seAFOod Polyp Prevention Trial is to determine whether the naturally-occurring ω-3 PUFA EPA, in the FFA form, prevents colorectal adenomas, either alone or in combination with aspirin.

The following primary hypotheses will be tested: i) EPA-FFA 2 g daily is more effective than placebo for reduction in adenoma recurrence; ii) Aspirin 300 mg daily is more effective than placebo for reduction in adenoma recurrence.

The secondary aim of the seAFOod Polyp Prevention Trial is to assess the tolerability and safety of EPA in the FFA form (EPA-FFA) alone and in combination with aspirin.

## Design

The seAFOod Polyp Prevention Trial is a randomized, double blind, placebo-controlled, 2 × 2 factorial trial of EPA-FFA 2 g daily and aspirin 300 mg daily.

### Setting

The Trial is integrated into the English BCSP such that participation in the Trial does not affect routine clinical care in either the screening or surveillance phase of the BCSP. The Trial was given Research Ethics Committee (REC) approval by the NRES Committee East Midlands (REC Reference 10/H0405/90). Approval for the Trial and subsequent substantial protocol amendments were also obtained from the English BCSP Research Committee.

The Trial is based in BCSP Centres, which comprise a variable number of individual BCSP endoscopy sites, at which patients are identified as ‘high risk’ and participants are randomised and followed-up prior to exit surveillance colonoscopy one year later.

Colonoscopy quality assurance (QA) in the BCSP is excellent [25]. Every BCSP colonoscopist must pass an accreditation examination and maintain a caecal intubation rate greater than 90% and an adenoma detection rate greater than 35%, thus minimising operator variability in Trial colonoscopy [25].

### Participants

BCSP patients between 55–73 years old, identified as ‘high risk’ (≥5 small adenomas or ≥3 adenomas, with at least one being ≥10 mm in diameter) at the first complete screening colonoscopy. Patients aged 74 years or above are excluded as they would not automatically be offered surveillance colonoscopy by the BCSP if the proposed examination occurred at an age in excess of 75 years of age.

Exclusion criteria are:

• Requirement for more than one repeat colonoscopy or flexible sigmoidoscopy within the BCSP 3-month screening window.

• Malignant change in an adenoma requiring Colorectal Cancer Multi-disciplinary Team management.

• Regular (>3 doses per week) prescribed or ‘over the counter’ (OTC) aspirin or regular (>3 doses per week) prescribed or OTC non-aspirin NSAID use.

• Aspirin intolerance or hypersensitivity, including aspirin-sensitive asthma.

• Active peptic ulcer disease within 3 months or previous peptic ulcer (not on proton pump inhibitor prophylaxis).

• Fish or seafood allergy.

• Current or planned regular (>3 doses per week) use of fish oil supplements.

• Known clinical diagnosis or gene carrier of a hereditary CRC predisposition (FAP, hereditary non-polyposis colorectal cancer).

• Previous or newly diagnosed inflammatory bowel disease.

• Previous or planned colorectal resection.

• Known bleeding diathesis or concomitant warfarin therapy or use of any other anti-coagulant or anti-platelet agent (e.g., Clopidogrel).

• Severe liver impairment.

• Severe renal failure (creatinine clearance <10 mL/min)

• Current methotrexate use at a weekly dose of 15 mg or more.

• Inability to comply with study procedures and agents.

• Serious medical illness interfering with study participation.

• Participant taking part in another interventional clinical trial.

• Failure to give written informed consent.

Note that prior use of NSAIDs or a fish oil preparation are not exclusions if they are self-prescribed, and not recommended by a doctor, and the patient is willing to stop the preparation for the duration of the Trial.

### Intervention

Participants are randomised to one of four groups according to a 2 × 2 factorial design in order to receive gastro-resistant EPA-FFA 2 g daily *per os* (as 2 × 500 mg ALFA™ capsules [SLA Pharma AG] taken twice daily with food) or identical placebo (capric and capryllic acid medium-chain triglycerides) [15] AND enteric-coated aspirin 300 mg daily *per os* (as one 300 mg tablet taken with food) or identical placebo until the day before surveillance colonoscopy (Table 1).

**Table 1:** seAFOod polyp prevention trial treatment allocation

	
Placebo	Placebo
EPA-FFA 1 g twice daily	Placebo
Placebo	Aspirin 300 mg once daily
EPA-FFA 1 g twice daily	Aspirin 300 mg once daily

Internet-based treatment assignment is determined by a computer-generated pseudo-random code using random permuted blocks of randomly varying size. Trial participants are allocated with equal probability to each treatment arm with stratification by BCSP Centre.

### Trial end-points

#### Primary end-point

The number of participants with one or more adenomas detected at the first BCSP surveillance colonoscopy.

#### Secondary end-points

i. The number of participants with one or more ‘advanced’ (≥10 mm diameter, high-grade dysplasia or tubulo-villous/villous histology) adenomas at the first BCSP surveillance colonoscopy.

ii. The number of ‘advanced’ adenomas per participant at the first BCSP surveillance colonoscopy.

iii. The total number of adenomas per participant at BCSP surveillance colonoscopy at the first BCSP surveillance colonoscopy.

iv. The region of the colorectum (right colon – any part of the colon proximal to the splenic flexure; left colon – the rectum and the colon distal to the splenic flexure) where adenomas are detected at the first BCSP surveillance colonoscopy.

v. The number of ‘high risk’ participants re-classified as ‘intermediate risk’ after the first BCSP surveillance colonoscopy (BCSP risk stratification at the first surveillance colonoscopy follows BSG Guidelines [23] so that any individual that does not continue to fulfil ‘high risk’ criteria is classified as ‘intermediate risk’ for further colonoscopic surveillance at three years).

vi. The number of participants with CRC detected prior to, or at, the first BCSP surveillance.

vii.  Adverse events, including clinically significant bleeding episodes.

#### Exploratory end-points

An important component of the seAFOod Polyp Prevention Trial is the measurement of levels of bioactive lipid mediators such as ω-3 PUFAs, 18R-HEPE, RvE1 and PGE-M in plasma, urine, erythrocytes and rectal mucosa in order to gain insight into the mechanism(s) of action of EPA and aspirin, alone and in combination, as well as to discover predictive biomarkers of EPA and/or aspirin chemoprevention efficacy. Laboratory biomarker studies are detailed in *The seAFOod Polyp Prevention Trial protocol for laboratory studies*, which is an Appendix to the main Trial protocol (http://www.eme.ac.uk/projectfiles/0910025protocol.pdf). All analyses will be conducted at the Good Clinical Laboratory Practice Yorkshire Experimental Cancer Medicine Centre laboratory, the Institute of Cancer Therapeutics in Bradford, and the Leeds Institute of Biomedical & Clinical Sciences.

### Trial schedule

**Figure 1:**
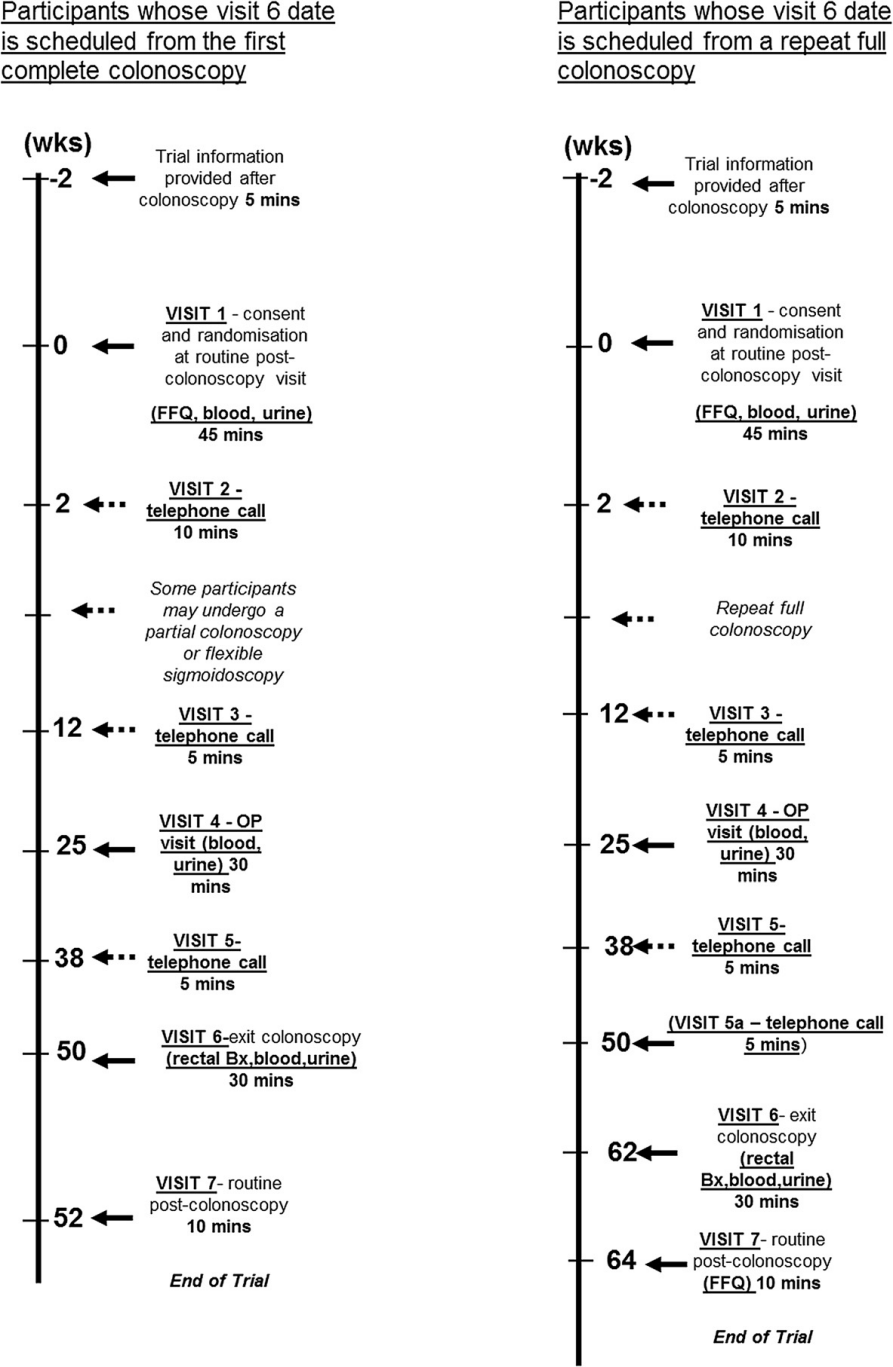
**Schedule of visits in the seAFOod Polyp Prevention Trial.** Trial interventions that are not part of routine BCSP care are in bold and underlined.

The detailed trial schedule is described in the full Trial Protocol at the EME programme website (http://www.eme.ac.uk/funded_projects/). Figure 1 is a summary of the trial visits and interventions.

In brief, ‘high risk’ BCSP patients are identified immediately at screening colonoscopy on the basis of adenoma number and (endoscopic) size, confirmed later by the histopathology report. Patients are formally assessed for eligibility by a BCSP Specialist Screening Practitioner (SSP) or Research Nurse (RN) when the histological adenoma size is available approximately 7–14 days after screening colonoscopy. If the patient is eligible, written informed consent is sought and obtained prior to randomisation. A validated Food Frequency Questionnaire (FFQ) is completed at baseline and at the end of the Trial so that any change in dietary ω-3 PUFA intake during Trial involvement can be determined [26]. Blood and urine are obtained at baseline (visit 1), 6 months (visit 4) and at (exit) surveillance colonoscopy (visit 6). Rectal biopsies are obtained at (exit) surveillance colonoscopy. Biological sample handling and storage are detailed in *The seAFOod Polyp Prevention Trial Manual of Biological Sample Standard Operating Procedures* (Additional file 1).

Participants receive Investigational Medicinal Product (IMP) in 6 month blocks at visit 1 and visit 4 in order to cover the 12 month period between screening colonoscopy and ‘high risk’ surveillance colonoscopy. It should be noted that participants who require a second colonoscopy within the stipulated three-month BCSP screening episode period require more than 12 months of IMP because the timing of the surveillance colonoscopy is usually set 12 months from the last complete examination of the colon (Figure 1). This is not relevant to participants who need a repeat flexible sigmoidoscopic examination or partial colonoscopy when the subsequent colonoscopy is still set 12 months from the Trial entry colonoscopy (Figure 1).

### Safety and tolerability considerations

Although it is not expected [9], increased bleeding risk associated with EPA-FFA alone, or in combination with aspirin, has not previously been evaluated in a large Phase III study. Therefore, we recommend that all participants stop IMP 10 days prior to, and for 4 days after, any invasive medical or surgical procedure taking place during the intervention period. Concomitant aspirin therapy alone is not stopped routinely for BCSP colonoscopy so that IMP can continue until the day of the (exit) surveillance colonoscopy.

Systematic review has not found any evidence of worsening of glycaemic control in diabetics taking omega-3 preparations [27], although there is a report of short-term loss of glycaemic control in diabetic patients taking 4 g EPA daily [28]. Trial participants with diabetes mellitus are reminded to monitor their glycaemic control by the usual means during the Trial.

Clinical studies indicate that EPA-FFA is well-tolerated at doses up to 2 g per day over periods up to 6 months [15]. The principal undesirable effects are expressed through the gastrointestinal tract with diarrhoea, abdominal pain, nausea and vomiting. These are normally relatively mild in severity and can be minimised or removed by dosing with food or dose reduction to 1 g daily [15]. If an adverse event is thought to be related to IMP, the dose of EPA-FFA or placebo can be temporarily reduced per protocol to 1 g per day aiming to increase the dose back to 2 g daily within 2 weeks.

### Sample size

The sample size estimate was based on the RCT of the same dose and preparation of EPA-FFA in FAP patients [15], a meta-analysis of aspirin RCTs [22] and detailed 2007–2008 BCSP audit data from the South of Tyne and Tees BCSP Centres.

In order to detect a minimum 18% relative reduction in adenoma risk in each two-arm comparison (less than the 22% reduction in polyp number compared with placebo in the FAP Trial [15] and below the absolute reduction in polyp number at one year [38%] in the aspirin RCTs [22]) from a 60% adenoma recurrence rate (South of Tyne and Tees BCSP data) at surveillance colonoscopy to 49%, 678 evaluable ‘high risk’ individuals need to be randomized equally to the four treatment arms, with 80% power at a 5% two-sided significance level. Standard practice for 2 × 2 factorial designs in the assumed absence of any interaction bases the sample size estimate on comparison ‘at the margins’ of active treatment A vs. placebo A (half of whom in each arm receive active treatment B and the other half placebo B). Assuming the same target effect size and alpha, this yields the same power for the marginal comparison of active treatment B vs. placebo B (half of whom in each arm receive active treatment A and the other half placebo A). With a total sample size of 678 for analysis, there is, in fact, a slight reduction in power (to 75%) which arises if *both* treatments work, because then the overall comparison for treatment A is not 0.49 vs. 0.6, but is 0.445 vs. 0.545 (averaging over the placebo and treatment B arms). To keep power at 80% for the above figures, 192 individuals are required per arm (total 768 evaluable ‘high risk’ individuals). Allowing for a 15% drop-out rate increases the sample size to 768/0.85 = 904 individuals.

Data from the South of Tyne and Tees BCSP Centres indicated that 20% of ‘high risk’ patients were expected to be using aspirin, and thus be ineligible for the trial. An allowance was made for an additional 20% of ‘high risk’ patients to be ineligible due to other reasons. Based on the assumption that 40% of patients would be ineligible it was calculated that a total of 904/0.6 = 1,507 ‘high risk’ patients would need to be identified at BCSP screening colonoscopy. Trial timelines were based on BCSP data suggesting that a BCSP Centre identifies approximately 50 ‘high risk’ patients per year. On this basis, the Trial intervention period was predicted to be 2 years and require 15 BCSP Centres (approximately 30 BCSP endoscopy sites).

### Statistical analysis

The main approach to analyses will be intention-to-treat (ITT), where the ITT population consists of all randomised participants. No adjustment for multiple significance testing was made in the sample size estimate or will be in the analyses. Results of comparative analyses will be presented as the appropriate point estimate (for example, risk ratio or difference in means), 95% confidence interval and *P* value. No formal interim analysis for efficacy is planned and hence there are no ‘stopping rules’.

#### Descriptive analyses

The baseline comparability of the groups will be assessed with regard to the following variables: age at randomization, gender, body mass index, cigarette smoking, alcohol consumption, diagnosis of diabetes, requirement for a repeat colorectal endoscopic procedure within 3 months, number of adenomas detected at baseline, number of ‘advanced’ adenomas detected at baseline and the location of adenomas in the colorectum. Categorical (including binary) variables will be summarized by reporting numbers and percentages in each category, continuous variables will be summarized using means and standard deviations. Missing data will be tabulated.

#### Primary analyses

For the primary outcome, it is anticipated that the 2 × 2 factorial trial will be analysed by an ‘at the margins’ approach, after first examining whether there is any evidence of an interaction between EPA and aspirin [29], although it is recognized that this will lack power to detect anything but a very large interaction effect. The log relative risk will be estimated using a log-binomial regression model with robust standard errors to allow for potential non-independence of observations within a BCSP centre. Both interventions will be fitted simultaneously and in a further secondary analysis, adjusted for ‘repeat colorectal endoscopic procedure within 3 months required’ plus any other covariates identified as important from the baseline comparisons. Should there be strong evidence of an interaction between EPA and aspirin, the effect of each treatment alone will be examined using an ‘inside the table’ approach, although it is recognized that precision for these analyses may be reduced since each uses only approximately 50% of the available sample.

A per-protocol analysis will be conducted as a sensitivity analysis, where the per-protocol population consists of all randomized participants who were not deemed to have a protocol violation. Additionally, some BCSP centres consist of multiple hospitals (sites), therefore an additional sensitivity analysis will be conducted in which both BCSP centre and site will be treated as random effects in a multi-level model.

#### Secondary analyses

All secondary end-points will be analysed using the ITT population, with the exception of adverse events, for which we will analyse the safety population, consisting of all participants who received at least one dose of trial medication.

• The relative recurrence of ‘advanced’ adenoma detected at the first BCSP surveillance colonoscopy will be analysed using a log-binomial regression model with robust standard errors to estimate the log relative risk.

• Number of ‘advanced’ adenomas per participant at the first BCSP surveillance colonoscopy will be analysed using a Poisson regression model with robust standard errors.

• Number of adenomas per participant at the first BCSP surveillance colonoscopy will be analysed using a Poisson regression model.

• The region of the colorectum where adenomas are detected at the first BCSP surveillance colonoscopy will be explored, possibly using a Poisson random effects model with bivariate response (corresponding to polyp counts in the left and right colon) in which treatment and a baseline polyp count will be independent variables together with random intercepts corresponding to patient and BCSP Centre.

• The number of ‘high risk’ participants re-classified as ‘intermediate risk’ after the first BCSP surveillance colonoscopy will be analysed using binary regression or logistic regression, treating centre as a random effect.

• The number of participants with CRC detected prior to or at the first BCSP surveillance will be analysed descriptively, and possibly using a logistic regression model depending on numbers with this outcome, although it is anticipated that there will be low power due to small numbers.

Adverse events, including clinically significant bleeding episodes will be summarized by tabulating the number (and percentage) of events occurring in each treatment arm. All participants who receive at least one dose of treatment will be included in the safety analysis. Mis-randomised participants will be analysed as treated. Adverse drug reactions (ADRs) will be summarized by severity using the preferred term and the worst severity and causality recorded. The worst case will be assumed if severity or causality are missing. All participants who experience treatment-emergent ADRs will be listed including participant identification, treatment arm, system organ class, preferred term, unexpectedness, seriousness, severity, start and stop dates/times, action taken and outcome.

## Discussion

The first patient first visit (FPFV) of the Trial was 11th November 2011. It soon became clear from screening logs at individual BCSP sites that the eligibility rate was 15–20% rather than the 60% that had been predicted in the original sample size calculation. One contributing factor was the use of other non-aspirin anti-platelet agents such as clopidogrel, which we later added as an extra exclusion criterion (Protocol version 3.1 dated 12 Jan 2012), in order to completely rule out the possibility of excess bleeding risk due to concomitant use of three agents with anti-platelet activity in one arm of the Trial. Another major contributing factor was the larger than expected number of ‘high risk’ individuals (approximately 25%) who required a repeat endoscopy, which was an exclusion criterion in earlier versions of the Trial protocol. We obtained data on the proportion of ‘high risk’ patients undergoing a repeat endoscopic procedure from participating BCSP sites and demonstrated that there had been a consistent increase in the number of patients undergoing repeat ‘re-look’ procedures since the start of the BCSP in 2006 and that ‘re-look’ rates varied significantly across BCSP sites. ‘High risk’ patients, who require a repeat screening procedure, were originally excluded from the Trial on the basis that the primary end-point might be confounded by adenoma detection and removal at an extra endoscopic procedure in-between the screening and one-year surveillance procedures. However, subsequent analysis (in April 2012) of the North-East BCSP Hub (9 BCSP Centres) and the Southern BCSP Hub (17 BCSP Centres) data on ‘high risk’ patients who underwent one-year surveillance colonoscopy in 2010 did not support this notion. Of 1,189 ‘high risk’ patients, 930 (78%) went straight to one-year surveillance after a single screening colonoscopy. One or more adenomas were detected in 465 (63%) of 738 patients, for whom data were available. The corresponding adenoma detection rate for ‘high risk’ patients, who underwent a repeat partial colonoscopy or flexible sigmoidoscopy within 3 months of the initial screening colonoscopy was 54% (59/110), but the adenoma detection rate in patients who underwent a repeat full colonoscopy was 67% (56/83). The overall adenoma detection rate at first surveillance colonoscopy was 62%, which is consistent with the value (60%) used in our original sample size calculation, although the cohort included patients who had undergone a repeat screening endoscopy. Therefore, we amended the protocol in order to allow recruitment of those individuals who require no more than one repeat endoscopic procedure (either colonoscopy or flexible sigmoidoscopy) within the 3-month screening episode window (Protocol version 4.0 dated 24 May 2012).

At the time of the latest sample size estimate in April 2012, we assumed that drop-out would be 10% rather than 15% (based on existing BCSP Trial site experience) and noted that 27 participants had already been recruited meaning that a further (768/0.9 = 853) – 27 = 826 participants were required. Data from the first 5 months’ recruitment experience and from the above 2010 BCSP Hub data indicated that approximately 24% of ‘high risk’ patients underwent a repeat endoscopy within 3 months of the first screening colonoscopy and that 27% of ‘high risk’ patients who were excluded because of the need for a repeat colorectal endoscopic procedure also met an additional exclusion criterion. Therefore, we estimated that the percentage recruitment could be increased from 15% (the current percent recruitment rate) to 15% + 17.5% (24% × 0.73) = 32.5%. Rounding the recruitment rate down to 30%, we estimated that the remaining number of ‘high risk’ individuals that would need to be screened from April 2012 to obtain 768 evaluable ‘high risk’ individuals is 827/0.3 = 2,757.

Revised Trial timelines are currently based on an overall ‘high risk’ patient recruitment rate of 30% and identification of approximately 50 ‘high risk’ patients by a BCSP Centre per year (this estimate has been confirmed by our data on the number of ‘high risk’ patients screened for the Trial by open, participating Centres, so far). In order to compensate, for the reduced percentage recruitment estimate, the number of participating BCSP Centres has been increased from 15 to 30 (equivalent to approximately 60 separate BSCP endoscopy sites throughout England).

The ability to recruit patients who have been identified as ‘high risk’ via FS screening, as well as by faecal occult blood test screening, should also increase the recruitment rate of the Trial, as the Bowel Scope programme is rolled out across England over the next two years.

The seAFOod Polyp Prevention Trial is the first Clinical Trial of an Investigational Medicinal Product (CTIMP) in the BCSP. The experience gained from the set-up and intervention phases of the Trial will be invaluable for the design of subsequent intervention trials in the BCSP. Early experience has confirmed that the BCSP is an excellent framework for a CRC chemoprevention RCT.

## Trial status (1/7/2013)

The Trial is active and recruiting patients from 59 BCSP sites (see Additional file 2).

Trial website: http://www.seafood-trial.co.uk/

The full Trial protocol is available at http://www.eme.ac.uk/funded_projects/

Trial Co-ordinating Centre

Nottingham Clinical Trials Unit, Nottingham Health Science Partners, C Floor South Block, Queens Medical Centre, Nottingham, NG7 2UH, United Kingdom

Phone: +44 (0) 115 8844931

Chief Investigator:

Professor Mark Hull

Principal Investigators:

Professor Colin Rees

Professor Richard Logan

Ms Gayle Clifford

Dr Paul Loadman

Professor Anna Nicolaou

Ms Diane Whitham

Trial Management Group:

Professor Mark Hull

Mrs Anna Sandell

Ms Diane Whitham

Professor Alan Montgomery

Dr Paul Loadman

Trial Steering Committee:

Professor Will Steward (Independent Chairman)

Professor Greg Rubin (Independent member)

Professor Stephen Halloran (Independent member)

Mr Alan Reece (PPI representative)

In attendance: Dr Chris Jordan (SLA Pharma AG), Dr Elmar Detering (Bayer Pharma AG), Professor Mark Hull, Professor Alan Montgomery, Professor Colin Rees and Professor Richard Logan

**Data Monitoring** &**Safety Committee:**

Professor Bob Steele

Professor Dion Morton

Professor John Norrie

Sponsorship, funding, ethics and registration details:

**Sponsor:** The University of Leeds (GA10/9312)

The Trial is funded by the Medical Research Council (MRC) and managed by the National Institute for Health Research (NIHR) within the EME Programme (reference number 09/100/25)

EudraCT number 2010-020943-10

REC reference number 10/H0405/90

**Clinical Trials Registration:**ISRCTN05926847

UKCRN Portfolio identification number 9734

## Competing interests

MAH has received an unrestricted scientific grant and conference travel expenses from SLA Pharma AG. MAH has received Consultancy fees from Bayer Pharma AG.

## Authors’ contributions

MAH is the Chief Investigator of the seAFOod Trial and leads the project, taking the main responsibility for preparing the protocol for journal publication. AS is the Trial Manager and DW is the Nottingham CTU Lead for the Trial. AM is the Trial Statistician. RFAL, GMC and CJR are Co-Investigators and have roles in the English BCSP. PML is the Lead Investigator for biological sample analysis in the Trial. All authors edited and approved the manuscript.

## Supplementary Material

Additional file 1 The seAFOod (Systematic Evaluation of Aspirin and Fish Oil) Polyp Prevention Trial. Click here for file

Additional file 2 The seAFOod Polyp Prevention Trial – Participating BCSP Endoscopy sites (1/7/2013). Click here for file
