# Supplementary material for: A randomized controlled trial of eicosapentaenoic acid and/or aspirin for colorectal adenoma prevention during colonoscopic surveillance in the NHS Bowel Cancer Screening Programme (The seAFOod Polyp Prevention Trial): study protocol for a randomized controlled trial
Source: Trials. 2013 Jul 29;14:237. doi: 10.1186/1745-6215-14-237 (PMC3733694; doi:10.1186/1745-6215-14-237)
Supplement: Additional file 1 — The seAFOod (Systematic Evaluation of Aspirin and Fish Oil) Polyp Prevention Trial. [file 1745-6215-14-237-S1.doc]

**
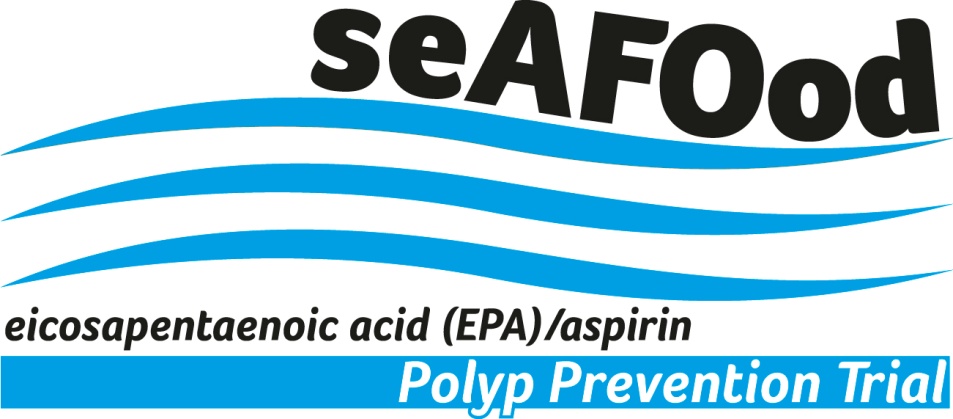
**

**The seAFOod (Systematic Evaluation of Aspirin and Fish Oil) Polyp Prevention Trial**

**Manual of Standard Operating Procedures for collection and storage of biological samples at BCSP Sites**

**TABLE OF CONTENTS**

1 contact details [3](#__RefHeading___Toc293992863)

2 introduction [4](#__RefHeading___Toc293992864)

3 scope [4](#__RefHeading___Toc293992865)

4 responsibilities [4](#__RefHeading___Toc293992866)

4.1 Bowel Cancer Screening Programme Centre staff [4](#__RefHeading___Toc293992867)

4.2 University of Bradford Laboratory staff [4](#__RefHeading___Toc293992868)

5 schedule of sample collection [5](#__RefHeading___Toc293992869)

6 consumables [5](#__RefHeading___Toc293992870)

6.1 Blood Samples [6](#__RefHeading___Toc293992871)

6.2 Urine Samples [6](#__RefHeading___Toc293992872)

6.3 Rectal Biopsies [6](#__RefHeading___Toc293992873)

6.4 Documentation and Labelling [6](#__RefHeading___Toc293992874)

7 collection, processing, storage and transportation of plasma, leukocytes and erythrocytes [7](#__RefHeading___Toc293992875)

7.1 Blood Sample Collection [7](#__RefHeading___Toc293992876)

7.2 Blood Sample Processing [7](#__RefHeading___Toc293992877)

7.3 Blood Sample Storage 9

8 collection, processing, storage and transportation of urine samples [9](#__RefHeading___Toc293992879)

8.1 Urine Sample Collection [9](#__RefHeading___Toc293992880)

8.2 Urine Sample Processing [9](#__RefHeading___Toc293992881)

8.3 Urine Sample Storage [9](#__RefHeading___Toc293992882)

9 collection, processing, storage and transportation of rectal mucosa [10](#__RefHeading___Toc293992883)

9.1 Rectal Biopsy [10](#__RefHeading___Toc293992884)

9.2 Mucosal Biopsy Processing [10](#__RefHeading___Toc293992885)

9.3 Mucosal Biopsy Storage [10](#__RefHeading___Toc293992886)

10 sample transportation [11](#__RefHeading___Toc293992887)

11 signatures [12](#__RefHeading___Toc293992888)

appendix one – Eppendorf 5702R Centrifuge [13](#__RefHeading___Toc293992889)

appendix two – Hanna H-141 Datalogger [14](#__RefHeading___Toc293992890)

# CONTACT DETAILS

| **seAFOod Polyp Prevention Trial Contacts** | | |
| --- | --- | --- |
|  | **Ms Anna Sandell**  **Trial Manager** | **Professor Mark Hull**  **Chief Investigator** |
| Address | Nottingham Health Science Partners  C Floor, South Block, Queen’s Medical Centre, Nottingham NG7 2UH | Wellcome Trust Brenner Building  Leeds Institute of Molecular Medicine  St James’s University Hospital  Leeds LS9 7TF |
| Tel: | 0115 8844931 | 0113 343 8650 |
| Fax: | 0115 9194430 | 0113 343 8702 |
| Email: | [Anna.sandell@nottingham.ac.uk](mailto:Anna.sandell@nottingham.ac.uk) | [M.A.Hull@leeds.ac.uk](mailto:M.A.Hull@leeds.ac.uk) |
| Role: | Trial Manager | Trial CI and Academic Co-Lead for laboratory studies |
|  | | |
| **seAFOod Polyp Prevention Trial Tissue Storage Facility** | | |
|  | **Dr Paul Loadman**  **Senior Lecturer in Pharmacokinetics** | **Professor Anna Nicolaou**  **Professor of Biological Chemistry** |
| Address | Institute of Cancer Therapeutics  University of Bradford  Tumbling Hill Street  Bradford BD7 1DP | School of Pharmacy  University of Bradford  Richmond Road  Bradford BD7 1DP |
| Tel: | 01274 233228 | 01274 234717 |
| Fax: | 01274 233234 | 01274 235600 |
| Email: | [P.M.Loadman@bradford](mailto:P.M.Loadman@bradford).ac.uk | A.Nicolaou@bradford.ac.uk |
| Role: | Academic Co-Lead for laboratory studies | Academic Co-Lead for laboratory studies |

| **seAFOod Polyp Prevention Trial Tissue Storage Facility** | | |
| --- | --- | --- |
|  | **Amanda Race**  **Research Assistant** |  |
| Address | Institute of Cancer Therapeutics  University of Bradford  Tumbling Hill Street  Bradford BD7 1DP |  |
| Tel: | 01274 235841 |  |
| Fax: | 01274 233234 |  |
| Email: | A.D.Race@bradford.ac.uk |  |
| Role: | Research Assistant |  |

# INTRODUCTION

The purpose of this Manual is to describe the collection, processing, storage and transportation of plasma, urine and rectal biopsy samples for biomarker measurement in the seAFOod Polyp Prevention Trial.

# SCOPE

This Manual is intended for use at participating Bowel Cancer Screening Programme Centre sites and at the central tissue storage facility at the University of Bradford.

All standard operating procedures (SOPs) described in this Manual are correct at the time of document finalisation and signature. Any update to the SOPs is the responsibility of the University of Bradford.

# responsibilities

## Bowel Cancer Screening Programme Centre staff

The staff at each participating Bowel Cancer Screening Programme Centre site, with support from the Trial Manager, are responsible for ensuring that plasma, leukocytes (white blood cells), erythrocytes (red blood cells), urine and rectal biopsies are collected, handled, processed and stored at the Bowel Cancer Screening Programme Centre site prior to transportation to the University of Bradford laboratory in accordance with the instructions in this Manual.

## 4.2 Institute of Cancer Therapeutics, University of Bradford Laboratory staff

Institute of Cancer Therapeutics laboratory staff, taking receipt of clinical samples from the Courier (CitySprint Healthcare), are responsible for checking and resolving any inconsistencies between samples received and the accompanying documentation, as described in the Standard Operating Procedure “SOP_SAMP_0005 Receipt and Storage of seAFOod Trial Samples”.

University of Bradford staff, with the support of the Trial Manager, will be responsible for

- Generating & issuing Clinical Laboratory Packs containing consumables
- Providing labels and sample worksheets required for each participant
- Supplying the latest version of this study laboratory manual
- Organising Courier transport for the samples (CitySprint Healthcare) from the Bowel Cancer Screening Programme Centre sites
- Tracking the collection, shipment (with the Courier), and receipt of all samples.

# schedule of sample collection

Please see the Trial Flow Diagram in the main Trial Protocol (Appendix B – Trial Schema). There are 7 clinical samples to be collected for analysis in the seAFOod Polyp Prevention Trial.

- Three blood samples (visit 1, pre-treatment; visit 4, mid-treatment, and visit 6, post-treatment) for plasma, leukocytes, and erythrocytes
- Three urine samples (visit 1, pre-treatment; visit 4, mid-treatment, and visit 6, post-treatment)
- Rectal biopsies from the exit (surveillance) colonoscopy (visit 6, post-treatment)

# CONSUMABLES

The majority of consumables will be supplied to the participating BCSP Centres by the University of Bradford, in the form of a Clinical Laboratory Pack (CLP). **Each CLP will contain sufficient consumables to collect and store the samples taken from one participant at one visit.** A collection of spare consumables will also be issued to the BCSP Centre. If extra/additional consumables are required for a participant visit, these should be taken from the spares provided, not from another CL pack. Each pack will contain:

| **Consumable** | **Supplier** | **Catalogue number** | **No. in Pack** | **Used for** |
| --- | --- | --- | --- | --- |
| K_2_ EDTA Vacutainers  (purple cap, 6ml draw volume) | BD Diagnostics | 367873 | 2 | Blood Collection |
| Disposable Pasteur Pipette 1ml | Sarstedt | 86.1172 | 5 | Transferring blood products to storage cryovials |
| Disposable Pasteur Pipette 3.5ml | Sarstedt | 86.1171 | 2 | Transferring urine to storage cryovials |
| 2ml Cryovials | Sarstedt | 72.379 | 9 | Storage of plasma, leukocytes, erythrocytes and biopsy (visit 6 only) samples |
| 5ml Cryovials | Sarstedt | 72.383 | 2 | Storage of Urine |
| Pathoseal Bag | Intelsius | - | 1 | Storage and transportation of cryovials |

BCSP Centres are expected to provide the equipment required for venepuncture (needles, syringes, tourniquets etc) and urine collection containers as per local Trust practice. The cost of these can be reimbursed by the University of Leeds on receipt of the appropriate invoice.

The University of Bradford will also provide each Site with a folder for storing completed sample worksheets prior to shipment and a “CyroRack 40” a storage system for bench-top use to ensure the cryovials are secure during the sample processing stage. A pen for labelling sample tubes and a pair of insulated gloves for placing tubes in dry ice (see Section 10) will also be provided. These items will be sent with the first shipment of CLPs.

## 6.1 Blood samples

Blood samples will be drawn directly into **two** K_2_ EDTA Vacutainers (purple cap, 6ml draw volume). Disposable Pasteur Pipettes (1ml) will be used to transfer plasma, leukocytes and erythrocytes aliquots into pre-labelled 2ml cryovials.

## 6.2 Urine samples

**Urine samples will be collected in a sterile collection container. Disposable Pasteur Pipettes (3.5ml) will be used to transfer urine into pre-labelled 5ml cryovials for storage.**

## 6.3 Rectal biopsies

Two rectal biopsies will be placed into each of two 2ml cryovials (total 4 biopsies) for immediate freezing.

## 6.4 Documentation and Labelling

A Sample worksheet should be completed for each participant at each visit when sampling takes place. This documents information needed by the Bradford laboratory. The sample worksheet should be completed using the appropriate sticky labels and stored in the trial folder until the samples are shipped to Bradford. The original sample worksheet should be sent with the samples and one carbon copy of the sample worksheet should be kept at the BCSP site and one carbon copy sent to the Clinical Trials Unit. More information on how to send the documentation is provided in section 10.

All cryovials used for sample storage must be labelled with the labels provided in the CRF before the contents have been added. These labels will document the Participant number, Participant’s initials, Visit and Sample Date, and are pre-numbered to identify the sample aliquot number. **All that needs to be written on each label is the participant initials after the participant ID number and the sample date.** An example of a plasma sample label is given below (for Participant #1 at site #1). Labels are colour-coded for separate specimens (plasma- pink; leukocytes-yellow; erythrocytes-red; urine-blue; rectal biopsies-green). Spare labels will also be provided. Labels should be completed (using the permanent waterproof pen provided or a similar one in your Department) and attached to the appropriate vials **before** samples are taken.


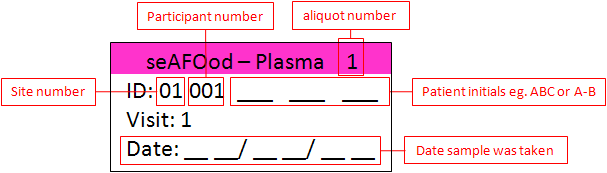


# collection, processing, storage and transportation of plasma, leukocytes and erythrocytes

## 7.1 Blood sample collection:

Blood samples will be obtained using the BD Vacutainer® system, following local Trust practice. Two purple cap K_2_ EDTA 6 ml tubes will be filled to capacity. Tubes should be agitated immediately to prevent clotting. The sample worksheet should be completed and a “worksheet” label attached from the prepared label sheet onto the top copy and 2 carbon copies of the worksheet

## 7.2 Blood sample processing:

Separate the blood components within 30 minutes of taking the blood sample. If not processed immediately, store the two K_2_EDTA Vacutainer tubes in the fridge at 4°C for up to 30 minutes.

Separation of blood components (Figure 1) is achieved by centrifugation of the sample in K_2_ EDTA Vacutainers in the refrigerated Eppendorf 5702R bench-top Centrifuge that has been provided for the Trial **or** your own Trust refrigerated Centrifuge if preferred. Centrifuge at 1,000 RCF (Relative Centrifugal Force) for 5 minutes at 4°C (Program #1 on the Eppendorf 5702R). Please note that the Eppendorf 5702R Centrifuge will need switching on a minimum of 20 minutes before use in order to get to the required temperature. Please see Appendix 1 for further instructions on how to use the Trial refrigerated Eppendorf 5702R bench-top Centrifuge. Complete the sample worksheet where appropriate.


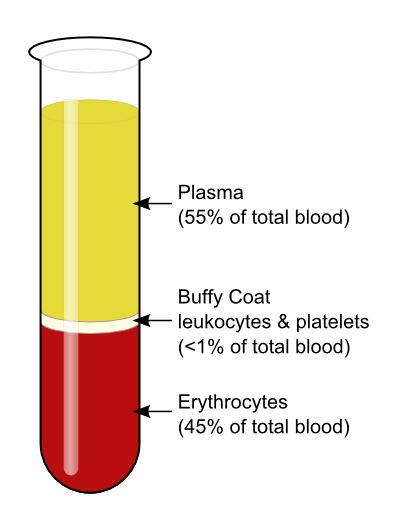


**Figure 1.** Centrifugation of an EDTA blood sample giving cell-free plasma, a ‘Buffy coat’ layer consisting of leukocytes (white blood cells) and platelets, and erythrocytes (red blood cells).

Specific labels provided for the Trial must be attached securely to each of **4** plasma cryovials (pink), **one** leukocyte cryovial (yellow) and **two** erythrocyte cryovials (red). These should be added prior to the samples being transferred to the vials as per section 6.4. The following information regarding the sample must be written on the vial labels using non-water soluble ink using the permanent waterproof pen provided or a similar one in your Department:

- **Participant initials after the site/participant number (see 6.4 above)**
- **Date of sample collection**

**Please check completion of each vial label carefully prior to adding the sample.**

Transfer the plasma (top clear layer) from each of the two blood sample Vacutainers into **two separate** **pre-labelled** 2ml cryovials (**total 4 cryovials per participant**), using a 1ml Pasteur pipette. All four cryovials should be filled with approximately equal amounts of plasma (usually between 0.5-1 ml). However, take care not to disturb the remaining leukocyte layer above the erythrocyte layer. Complete the sample worksheet.

If any of the red blood cell pellet is carried across, return the pipetted plasma into the vacutainer and re-spin the plasma samples as above and transfer the plasma to a fresh cryovial.

Next, remove the remaining leukocyte/platelets layer (approximately 1 ml) from the two Vacutainer tubes and place into **one** **pre-labelled** 2 ml cryovial using a clean pipette but the same pipette can be used to transfer both layers into this cryovial. It does not matter if some contaminating erythrocytes, bottom layer, are present in this sample. Complete the sample worksheet.

Finally, using a clean pipette, transfer the erythrocyte (red) layer from one of the Vacutainers in two approximately equal amounts into **two separate** **pre-labelled** 2 ml cryovials (total 2 cryovials per participant). Complete the sample worksheet.

Dispose of the Vacutainers and used Pasteur pipettes as per local Trust practice for disposal of biological samples and contaminated equipment.

The details of the samples must be recorded on the sample worksheet provided in the CLP. The original sampling worksheet, containing the details of the samples to be shipped, should be kept in the Ring Binder provided for completed worksheets until the samples are shipped, when the completed worksheets will be sent with the shipment. **One carbon copy of the sample worksheet should be kept at the BCSP Centre site and one carbon copy should be sent to the Clinical Trials Unit.**

## 7.3 Blood sample storage:

Place all the cryovials derived from the blood sample in the Pathoseal Bag provided in the CLP and transfer to the Trial Liebherr Underbench -20^o^C freezer or your existing Trust -20^o^C freezer immediately. Please see Appendix 2 for details of how to use the Hanna HI-141 CH Datalogger inside your -20^o^C freezer. Do not seal the Pathoseal Bag until it contains all the samples (blood components, urine and biopsy if applicable) have been taken at the visit for this participant. The Pathoseal Bag will be stored in the -20°C freezer until shipment to the Central Trial Laboratory in Bradford. Label the bag with the participant identification details. Check that the sample worksheet has been completed and labelled properly.

# COLLECTION, PROCESSING, STORAGE AND TRANSPORTATION OF URINE SAMPLES

## Urine sample collection:

A urine sample of approximately 10 ml is required.

## Urine sample processing:

Specific labels (blue) provided for the Trial must be added and fastened securely to each of 2 urine cryovials prior to transferring the sample into the vial. The following information regarding the sample must be written on the vial labels using non-water soluble ink using the permanent waterproof pen provided or a similar one in your Department:

- **Participant initials after the site/participant number (see 6.4 above)**
- **Date of sample collection**

**Please check completion of each cryovial label carefully prior to adding the sample.**

Transfer approximately 5 ml urine from the collection container into two separate 5ml **pre-labelled** cryovials using a 3.5ml Pasteur pipette. Complete the sample worksheet .

Dispose of the remaining urine and used Pasteur pipettes as per local Trust practice for disposal of biological samples.

The details of the samples must be recorded on the sample worksheet provided in the CLP. The original sample worksheet, containing the details of the samples to be shipped, should be kept in the Ring Binder provided for completed worksheets until the samples are shipped, when the completed worksheets will be sent with the shipment. **One carbon copy of the sample worksheet should be kept at the BCSP centre and one carbon copy should be sent to the Clinical Trials Unit**

## Urine sample storage

Urine sample cryovials should be placed in the Pathoseal Bag provided in the CL pack and transferred to the -20°C freezer immediately following sample processing. Complete and label the sample worksheet. Do not seal the Pathoseal Bag until it contains all the samples (blood components, urine and biopsy if applicable) have been taken at the visit for this participant. The Pathoseal Bag will be stored in the -20°C freezer until shipment to the Central Trial Laboratory in Bradford. Ensure the bag is labelled with the participant identification details.

# COLLECTION, PROCESSING, STORAGE AND TRANSPORTATION OF RECTAL MUCOSA

## Rectal biopsy:

Four rectal biopsies will be taken randomly from the posterior wall of the rectum at the end of the 12 month surveillance colonoscopy (visit 6, post-treatment) using 6 mm jaw forceps. Biopsies should be obtained from macroscopically normal mucosa at least 2 cm from a polypectomy site.

## Mucosal biopsy processing:

Specific labels provided for the Trial must be added and fastened securely to each of 2 cryovials prior to transferring the sample into the vial. The following information regarding the sample must be written on the vial labels using non-water soluble ink using the permanent waterproof pen provided or a similar one in your Department:

- **Participant initials after the site/participant number (see 6.4 above)**
- **Date of sample collection**

Two biopsies will be placed immediately into each of **two** **pre-labelled** 2 ml cryovials. Complete the sample worksheet. The original sampling worksheet, containing the details of the samples to be shipped, should be kept in the Ring Binder provided for completed worksheets until the samples are shipped, when the completed worksheets will be sent with the shipment. **One carbon copy of the sample worksheet should be kept at the BCSP centre and once carbon copy should be sent to the Clinical Trials Unit**

## Mucosal biopsy storage:

The cryotubes containing rectal mucosa should be placed **immediately** in the Pathoseal Bag and transferred to the -20°C freezer. Complete and label the sample worksheet. Do not seal the Pathoseal Bag until it contains all the samples (blood components, urine and biopsy) have been taken at visit 6 for this paticipant. The Pathoseal Bag will be stored in the -20°C freezer until shipment to the Central Trial Laboratory in Bradford. Ensure the bag is labelled with the participant identifier.

# sample transportation

University of Bradford staff, in close collaboration with the Trial Manager, will organise pick-up and transportation of all samples from each BCSP Centre site approximately every three months by specialist Courier (CitySprint Healthcare).

BCSP Centre sites have been grouped into individual routes by CitySprint Healthcare with collection from these sites being on the same day.

The day before collection, CitySprint Healthcare will arrange for thermal transportation units containing dry ice to be sent to BCSP unit. The samples can be transferred to the thermal transportation unit on the delivery date, or on the collection date, allowing flexibility for the BCSP Centre personnel. The thermal transportation unit can be kept at room temperature and can be stored in a standard endoscopy room until collection. The lid of the thermal transportation unit should only be taken off when necessary for adding the samples.

All sealed Pathoseal bags collected since the last Courier sample collection should be placed in the thermal transportation unit. Dry ice should be spread around the bags to ensure they are completely covered. Please remember that dry ice is very cold and can cause cold burns. **Use gloved hands to spread the dry ice** (or other suitable implement). Care should be taken not to have prolonged contact with the dry ice. A shipment form documenting the samples being transported must be labelled and completed. The carbon copy of the shipment form should be kept in the Ring Binder with the completed sample worksheets The original shipment form, along with the completed original sample worksheets for all the samples, must be sent with the thermal transportation unit (but kept away from the samples and dry ice). The thermal transportation unit will be pre-labelled with the address and contact details for the University of Bradford staff and the words “**GCLP laboratory clinical samples**”.

On receipt of each shipment of samples in Bradford, the procedures described in University of Bradford Standard Operating Procedure “SOP_SAMP_0005 Receipt and Storage of seAFOod Trial Samples” will be followed. Each consignment will be checked to ensure that copies of all the relevant sampling worksheets are enclosed. If there is any discrepancy between samples recorded on a sample worksheet & samples included in transportation, the person responsible for despatching the shipment will be contacted in order to resolve inconsistencies.

**11 SIGNATURES**

| I confirm that the information in this Laboratory Manual has been reviewed by the Laboratory personnel involved in the sample analysis and by the Trial Team. This document does not exceed or contradict the requirements set out in the full Clinical Protocol. | | | | |
| --- | --- | --- | --- | --- |
|  | | | | |
|  | Name: | Professor Mark Hull | Date: 24/11/2011 |  |
|  | Title: | Chief Investigator |  |  |
|  | Signature: | 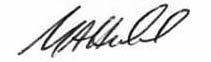 |  |  |

|  | | | | |
| --- | --- | --- | --- | --- |
|  | | | | |
|  | Name: | Dr Paul Loadman | Date: 24.11.2011 |  |
|  | Title: | Co-Investigator |  |  |
|  | Signature: | 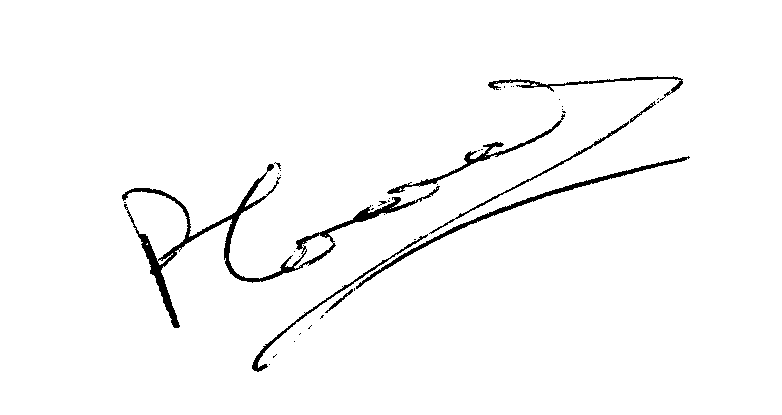 |  |  |

|  | | | | |
| --- | --- | --- | --- | --- |
|  | | | | |
|  | Name: | Professor Anna Nicolaou | Date: 24.11.2011 |  |
|  | Title: | Co-Investigator |  |  |
|  | Signature: |  |  |  |

# Appendix one

**USE OF THE EPPENDORF 5702R REFRIGERATED BENCH-TOP CENTRIFUGE**

This Centrifuge has been chosen for ease of use. It is refrigerated so that the samples remain as cold as possible during centrifugation because some of the substances that we will measure are labile at room temperature.

Detailed information is available in the Eppendorf Centrifuge 5702R Instruction Manual.

The Centrifuge will be pre-programmed by the Engineer who delivers the Centrifuge. **Program #1 is the only setting that is required for the seAFOod Trial blood samples.**

1. Turn the Centrifuge on at least 20 minutes before the expected time that it is needed to centrifuge one or more samples.
2. Make sure that the temperature is set at 4^o^C and the lid is closed.
3. Approximately 15-20 minutes later, check that the internal temperature has reached 4^o^C.
4. When the internal temperature has reached 4^o^C, place the blood sample tubes to be centrifuged into the tube buckets so that the tubes are symmetrically placed around the rotor (to ensure the Centrifuge is balanced). If there is/are an odd number of sample tubes, please place a ‘balance’ tube filled with approximately the same amount of water as blood in the appropriate bucket.
5. Close the Centrifuge lid.
6. Press PROG 1 button. This is a program that spins the tubes at 1,000 RCF (Relative Centrifugal Force) for 5 minutes.
7. At the end of the Centrifuge cycle, remove the tubes taking care to not disturb the contents. Aliquot plasma, leukocytes and the red blood cell layers as described on pages 7-8.
8. If you can still see red blood cells in the “plasma” layer repeat steps 4 and 7 again.

# Appendix two

**USE OF THE HANNA H-141 CH DATALOGGER IN THE -20 FREEZER CONTAINING seAFOod TRIAL SAMPLES**

The Hanna H-141 CH Datalogger is a white plastic instrument that monitors the temperature in the freezer so that we can confirm that the samples have been stored properly between taking the samples and collection by the Courier.

The Datalogger is supplied with a Lithium battery which will have a life longer than the expected duration of the Trial. The H141000 application software will be installed on a PC of your choice by the Engineer who delivers the equipment. Otherwise follow the instructions in order to install yourself. Parameters for acquiring temperature data will be pre-set at a 5 minute interval.

After the Liebherr Underbench -20^o^C freezer has been installed or an alternative -20^o^C freezer has been identified for Trial sample storage, simply place the instrument on the top shelf of the freezer.

Each time anyone goes in the freezer, the temperature on the LCD should be noted. If it is above -15^o^C, please download the temperature data immediately (see below) and send the MS-Excel file to Amanda Race a.d.race@bradford.ac.uk naming the file with site name_DD_MM_YY.xl.

If the temperature is always below -15^o^C, routine download of temperature data should be performed every 6 months, for which you will receive a prompt by e-mail.

Instructions for downloading data

1. Connect the H141001 Infrared transmitter to the computer via a RS232 port (extension cable supplied with datalogger)
2. Remove the datalogger from the freezer and place on the transmitter, taking care to align the ribs on the logger with the slots on the transmitter
3. Open the acquisition software and save the downloaded data as a MS-Excel file naming the file with site name_DD_MM_YY.xl.
4. Send the Excel file by e-mail to Amanda Race a.d.race@bradford.ac.uk
