# Supplementary material for: A randomized controlled trial of eicosapentaenoic acid and/or aspirin for colorectal adenoma prevention during colonoscopic surveillance in the NHS Bowel Cancer Screening Programme (The seAFOod Polyp Prevention Trial): study protocol for a randomized controlled trial
Source: Trials. 2013 Jul 29;14:237. doi: 10.1186/1745-6215-14-237 (PMC3733694; doi:10.1186/1745-6215-14-237)
Supplement: Additional file 2 — The seAFOod Polyp Prevention Trial – Participating BCSP Endoscopy sites (1/7/2013). [file 1745-6215-14-237-S2.docx]

**The seAFOod Polyp Prevention Trial – Participating BCSP Endoscopy sites (1/7/2013)**

Airedale NHS Trust

**Basildon** and Thurrock University **Hospitals NHS** Foundation **Trust**

**Blackpool**, Fylde and Wyre Hospitals **NHS Trust**

Bradford Teaching Hospitals NHS Foundation Trust

Calderdale and Huddersfield NHS Foundation Trust (Halifax and Huddersfield)

Chesterfield Royal Hospitals NHS Foundation Trust

City Hospitals Sunderland NHS Foundation Trust

**Colchester** Hospital University **NHS** Foundation **Trust**

County Durham and Darlington NHS Foundation Trust

Derby Hospitals NHS Foundation Trust

Dorset County Hospital **NHS** Foundation **Trust (Dorchester)**

East and North **Hertfordshire NHS Trust (Welwyn Garden City)**

**Frimley Park** Hospital **NHS** Foundation **Trust**

Gateshead Health NHS Foundation Trust

**George Eliot** Hospital **NHS Trust (Nuneaton)**

**Gloucestershire** Hospitals **NHS** Foundation **Trust (Gloucester and Cheltenham)**

**Hinchingbrooke** Health Care **NHS Trust**

Hull & East Yorkshire Hospitals NHS Trust

**Ipswich** Hospital **NHS Trust**

Kettering General Hospital NHS Foundation Trust

**King's College Hospital NHS** Foundation **Trust**

Lancashire Teaching Hospitals **NHS** Foundation **Trust (Preston)**

Leeds Teaching Hospitals NHS Trust

**Lewisham Hospital NHS Trust**

Mid Yorkshire Hospitals NHS Trust (Wakefield)

Newcastle Upon Tyne Hospitals NHS Foundation Trust

Norfolk & Norwich University Hospital NHS Foundation Trust

North Bristol **NHS Trust**

North Cumbria University Hospitals NHS Trust (Carlisle)

**Northampton** General Hospital **NHS Trust**

Northern Lincolnshire and Goole Hospitals NHS Trust (Grimsby and Scunthorpe)

North Tees and Hartlepool NHS Foundation Trust (Stockton and Hartlepool)

Northumbria Healthcare NHS Trust (North Tyneside and Wansbeck)

Nottingham University Hospitals NHS Trust

**Peterborough** and Stamford Hospitals **NHS** Foundation **Trust**

**Plymouth** Hospitals **NHS Trust**

**Poole** Hospital **NHS** Foundation **Trust**

**Royal Bolton** Hospital **NHS** Foundation **Trust**

**Royal Bournemouth** and Christchurch Hospitals **NHS** Foundation **Trust**

**Royal Cornwall** Hospitals **NHS Trust**

**Royal Surrey** County **NHS** Foundation **Trust**

Royal **Wolverhampton NHS Trust**

Sherwood Forest Hospitals **NHS** Foundation **Trust (Mansfield)**

South Devon Healthcare **NHS** Foundation **Trust (Torquay)**

South Tyneside NHS Foundation Trust

South Warwickshire NHS Foundation Trust

Taunton and Somerset **NHS** Foundation **Trust**

University Hospitals **Bristol NHS** Foundation **Trust**

University Hospitals Coventry and Warwickshire NHS Trust

University Hospitals of Leicester NHS Trust (Glenfield)

University Hospitals of Morecambe Bay NHS Foundation Trust (Kendal)

**West Hertfordshire** Hospitals **NHS Trust (Hemel Hempstead)**

**Yeovil** District Hospital **NHS** Foundation **Trust**

York Hospitals NHS Trust
